# Electronic waste – an emerging threat to the environment of urban India

**DOI:** 10.1186/2052-336X-12-36

**Published:** 2014-01-20

**Authors:** Santhanam Needhidasan, Melvin Samuel, Ramalingam Chidambaram

**Affiliations:** 1 Saveetha School of Engineering, Saveetha University, Chennai, Tamil Nadu 602 105, India; 2 School of Biosciences and Technology, VIT University, Vellore, Tamil Nadu, India

**Keywords:** Chemical leaching, Biological leaching, E-waste, Environmental hazard, Occupational hazard, Toxic

## Abstract

Electronic waste or e-waste is one of the emerging problems in developed and developing countries worldwide. It comprises of a multitude of components with valuable materials, some containing toxic substances, that can have an adverse impact on human health and the environment. Previous studies show that India has generated 0.4 million tons of e-waste in 2010 which may increase to 0.5 to 0.6 million tons by 2013–2014. Coupled with lack of appropriate infrastructural facilities and procedures for its disposal and recycling have posed significant importance for e-waste management in India. In general, e-waste is generated through recycling of e-waste and also from dumping of these wastes from other countries. More of these wastes are ending up in dumping yards and recycling centers, posing a new challenge to the environment and policy makers as well. In general electronic gadgets are meant to make our lives happier and simpler, but the toxicity it contains, their disposal and recycling becomes a health nightmare. Most of the users are unaware of the potential negative impact of rapidly increasing use of computers, monitors, and televisions. This review article provides a concise overview of India’s current e-waste scenario, namely magnitude of the problem, environmental and health hazards, current disposal, recycling operations and mechanisms to improve the condition for better environment.

## Introduction

Electronic gadgets are meant to make our lives happier and simpler, but they contain toxic substances, their disposal and recycling becomes a health nightmare. It has penetrated every aspect of our lives and most of us do not think about what happens to these gadgets when we discard or upgrade. The use of electronic devices has proliferated in recent decades and proportionality, the quantity of electronic devices that are disposed of, is growing rapidly throughout the world. E-waste is an emerging problem given the volumes of e-waste being generated and the content of both toxic and valuable materials in them. This fast growing waste stream is accelerating because the global market for personal computers (PC) is far from saturation and the average life span of a PC is decreasing rapidly. The life span of central processing units (CPU) had reduced from 4–6 years in 1997 to 2 years in 2005 [1-5]. Over the past two decades, the global market of electrical and electronic equipment (EEE) continues to grow exponentially, while the life span of those products becomes shorter and shorter. Predictably, the number of electrical devices will continue to increase on the global scale, and microprocessors will be used in ever increasing numbers in daily objects. The production of EEE is one of the fastest growing global manufacturing activities. Rapid economic growth, coupled with urbanization and a growing demand for consumer goods, has increased both the consumption and the production of EEE [6-8].

This new kind of waste is posing a serious challenge in disposal and recycling to both developed and developing countries. While having some of the world’s most advanced high-tech software and hardware developing facilities, India’s recycling sector can be called medieval [9]. The dumping of e-waste, particularly computer waste, into India from developed countries and all this has made e-waste management an issue of environment and health concern. Compared to conventional municipal wastes, certain components of electronic products contain toxic substances, which can generate a threat to the environment as well as to human health [5,10-12]. For instance, television and computer monitors normally contain hazardous materials such as lead, mercury, and cadmium, while nickel, beryllium, and zinc can often be found in circuit boards. Due to the presence of these substances, recycling and disposal of e-waste becomes an important issue.

Most people are unaware of the potential negative impact of the rapidly increasing use of computers, monitors, and televisions. When these products are placed in landfills or incinerated, they pose health risks due to the hazardous materials they contain. The improper disposal of electronic products leads to the possibility of damaging the environment. As more e-waste is placed in landfills, exposure to environmental toxins is likely to increase, resulting in elevated risks of cancer and developmental and neurological disorders. A major driver of the growing e-waste problem is the short life span of most electronic products—less than two years for computers and cell phones [13,14]. In a 2006 report, the International Association of Electronics Recyclers projected that, with the current growth and obsolescence rates of the various categories of consumer electronics, somewhere in the neighborhood of 3 billion units would be scrapped by 2010 or an average of about 400 million units a year.

This review article provides a concise overview of India’s current e-waste scenario, namely magnitude of the problem, environmental and health hazards, current disposal and recycling operations.

### Electronic waste

Electronic waste commonly known as e-waste is the popular name given to electronic products nearing or at the end of its useful life. E-waste in short is a generic term embracing various forms of electric and electronic equipment that have ceased to be of any value to their owners. Puckett et al. define E-waste as “a broad and growing range of electronic devices ranging from large household devices such as refrigerators, air conditions, cell phones, personal stereos, and consumer electronics to computers which have been discarded by their users”. According to Sinha-Khetriwal, “E-waste can be classified as any electrical powered appliance that has reached its end-of-life”. As there does not seem to be a standard definition for E-waste, the definition offered by Sinha-Khetriwal et al. can be adopted for this paper. It is comprised of discarded computers, television (TV) sets, mobile phones, microwave ovens and other such appliances that are past their useful lives.

The composition of e-waste is very diverse and differs in products across different categories. It contains more than 1000 different substances, which fall under ‘hazardous’ and ‘non-hazardous’ categories. Broadly, it consists of ferrous and non-ferrous metals, plastics, glass, wood and plywood, printed circuit boards (PCB), concrete and ceramics, rubber and other items. Iron and steel constitute about 50% of the E-waste followed by plastics (21%), non-ferrous metals (13%) and other constituents. Non-ferrous metals consist of metals like copper (Cu), aluminum (Al) and precious metals, e.g. silver (Ag), gold (Au), platinum, palladium, etc. The presence of elements like lead, mercury, arsenic, cadmium, selenium and hexavalent chromium and flame retardants beyond threshold quantities of e-waste classifies them as hazardous waste [11-13].

### Global scenario of e-waste

Quantity of E-waste generated and the content of toxic and valuable materials, it has become an emerging problem throughout the world. In 1994, it was estimated that approximately 20 million that is about 7 million tons of PCs became obsolete. In 2010 this figure has increased to over 150 million PCs. Over the past two decades, the global market of EEE continues to grow exponentially, while the lifespan of those products becomes shorter and shorter. In the United States (US) market, less than 80 million communication devices were sold in 2003, 152 million by 2008, a growth of over 90 percent in 5 years and by 2015 this numbers would be skyrocketing. Meanwhile, in 2006, more than 34 million TVs have been exposed in the market, and roughly 24 million PCs and 139 million portable communication devices have been produced. In the European Union (EU), the total units of electronic devices placed on the market in 2009 were more than 3.8 billion units, including 265 million computers, roughly 245 million in home consumer electronics, and 197 million consumer appliances. In China, approximately 20 million refrigerators and more than 48 million TVs were sold in 2001, and nearly 40 million PCs were sold in 2009. The situation is exacerbated by the rapid turnover of electronic devices. Because of the fast pace at which technology is evolving, most electronics have only a 2 to 3 year useful life. Apple sells more than 300,000 new phones every day in the world market and in this same time frame, more than 150,000 new Blackberries are also sold and 700,000 new Android phones are being activated. Most of the phones that are replaced by these new devices end up in a draw or in municipal landfills [15-17].

Electronic waste has raised concerns because many components in these products are toxic and are not biodegradable. Based on these concerns, many European countries banned E-waste from landfills long before in the 1990s. Alarming levels of dioxin compounds, linked to cancer, developmental defects, and other health problems in the samples of breast milk, placenta, and hair, these compounds are linked to improper disposal of electronic products. Furthermore, surveys have indicated that much exported, E-waste is disposed of unsafely in developing countries, leaving an environmental and health problem in these regions. Impacts from those countries, especially Asia, have already been reported. Meanwhile, recycling and disposal of E-waste are also grown in the regions beyond Asia, particularly in certain African countries. Today’s paradigm is one of disposable electronics, and as a result we now stand at the forefront of a growing environmental catastrophe [18-21].

### Problem in urban India

The Indian information technology (IT) industry has been one of the major drivers of change in the economy in the last decade and has contributed significantly to the digital revolution being experienced by the world. New electronic gadgets and appliances have infiltrated every aspect of our daily lives, providing our society with more comfort, health and security and with easy information acquisition and exchange. India has generated about 0.2 million tons of E-waste in 2006 and in 2010 it is about 0.4 million tons and at present the quantum is increasing rapidly. Studies so far reveal that the total e-waste generation in India from both households and corporate will reach 0.5 to 0.6 million tons by 2013–2014 [11].

### Personal computer penetration in India

**Figure 1:**
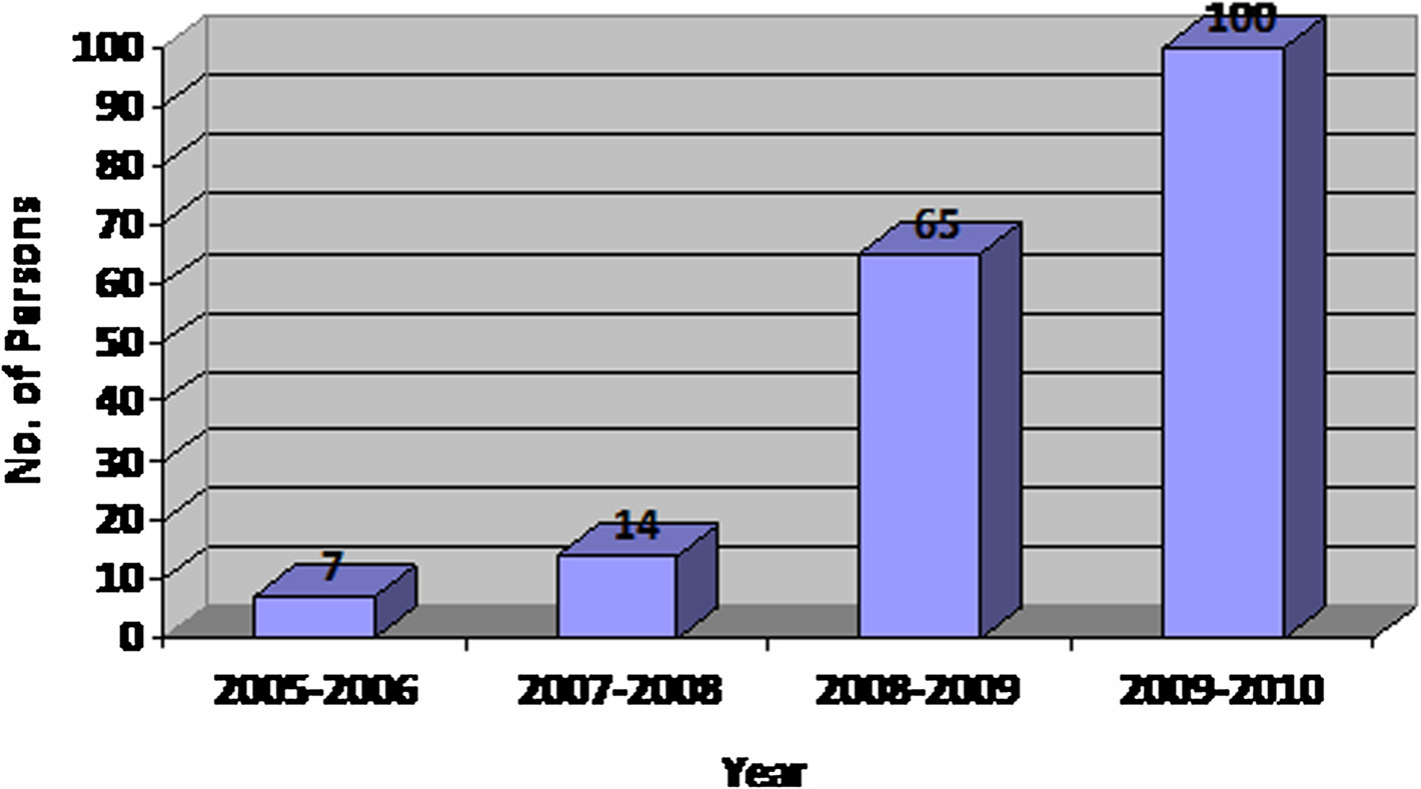
Usage of PCs for every 1000 persons.

Penetration of personal computers in India has increased drastically in the recent years. The following Figure 1, shows the usage of personal computers for every 1000 persons increases year after year.

**Figure 2:**
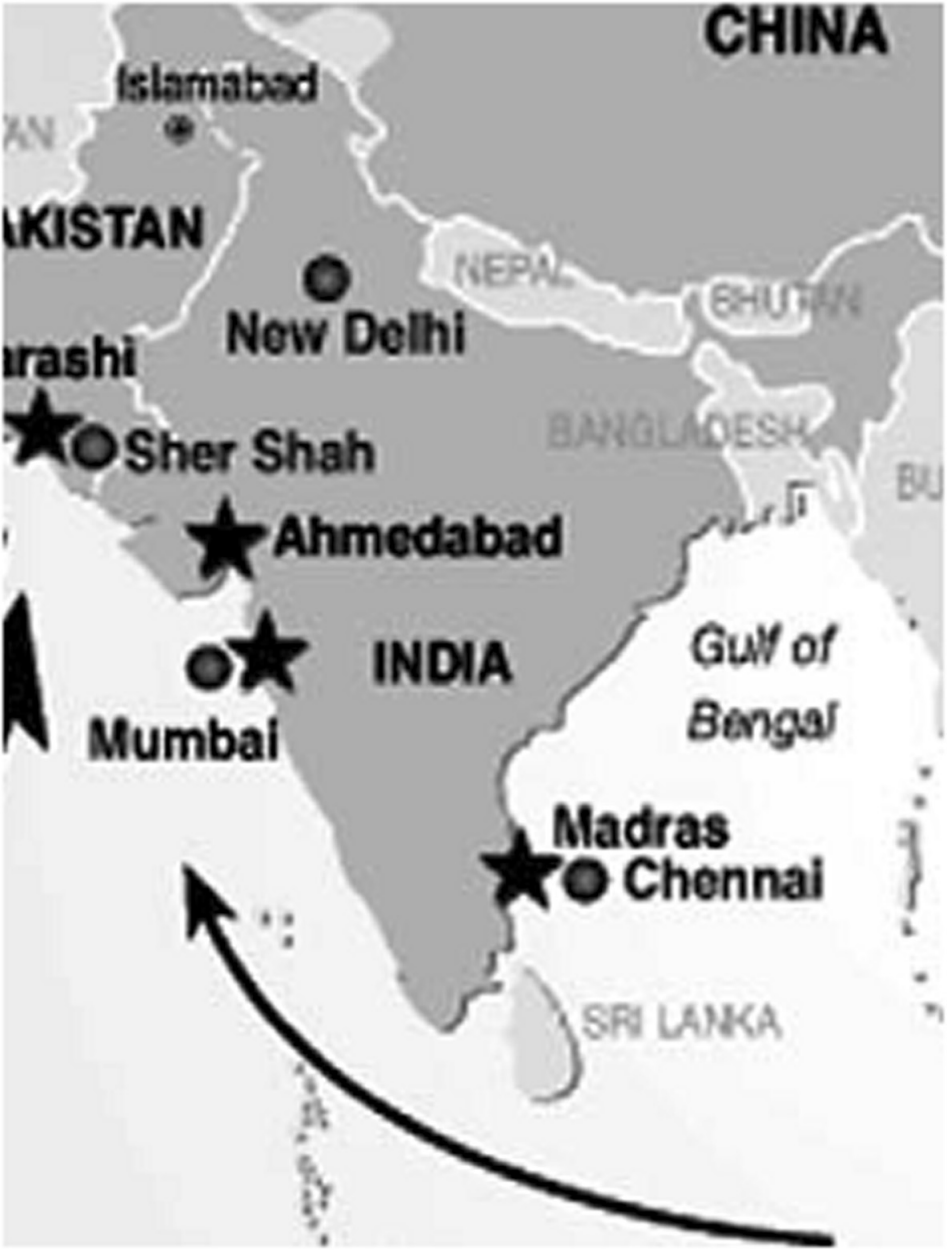
Major Indian ports receiving E-waste.

Of the total e-waste generated in the country, western India accounts for the largest volume at 35%, while the southern, northern and eastern regions account for 30, 21 and 14%, respectively. The top states in the order of highest contribution to waste electrical and electronic equipment (WEEE) include Maharashtra, Andhra Pradesh, Tamil Nadu, Uttar Pradesh, West Bengal, Delhi, Karnataka, Gujarat, Madhya Pradesh and Punjab. The Table 1, gives the total WEEE generation in the State of Maharashtra. Figure 2, shows the major Indian ports which receives E-waste in large from other countries as well. The city-wise ranking of the largest WEEE generators is Mumbai, Delhi, Bangalore, Chennai, Kolkata, Ahmedabad, Hyderabad, Pune, Surat and Nagpur.

**Table 1:** Total WEEE generation in the State of Maharashtra

**S. no**	**Place**	**Quantity of generation (tonnes)**
1	Navi Mumbai	4636.96
2	Greater Mumbai	11,017.06
3	Pune	3584.21
4	Pimpri-Chinchwad	1032.37
	**Total**	**20,270.60**

An estimated 30,000 to 40,000 computers become obsolete every year from the IT industry in Bangalore alone. Home to more than 1200 foreign and domestic technology firms, Bangalore figures prominently in the danger list of cities faced with an e - waste hazard. As much as 1000 tons of plastics, 300 tons of lead, 0.23 ton of mercury, 43 tons of nickel and 350 tons of copper are annually generated in Bangalore. While on the basis of scrap handled by the Delhi-based scrap dealers, their total number of PCs meant for dismantling would be around 15,000 per year. This figure does not include PCs handled by large dealers who get scraps from foreign sources. Mumbai, the financial nerve-center of India, alone throws away 19,000 to 20,000 tons of electronic waste a month, excluding the large e-waste it imports from developing nations through its port [12]. There are only two formal recyclers one at Chennai and another in Bangalore for the whole of South India and one in western India. Currently, there are no formal recyclers operating in the north or the east. Over 1 million poor people in India are involved in the manual recycling operations of E-waste and most of the people working in this recycling sector are the urban poor with very low literacy levels and hence very little awareness regarding the hazards of e-waste toxins. There are a sizeable number of women and children who are engaged in these activities and they are more vulnerable to the hazards of this waste. The following three categories of WEEE account for almost 90% of the generation:

▪ Large household appliances: 42%,

▪ Information and communications technology equipment: 33.9% and

▪ Consumer electronics: 13.7%.

**Figure 3:**
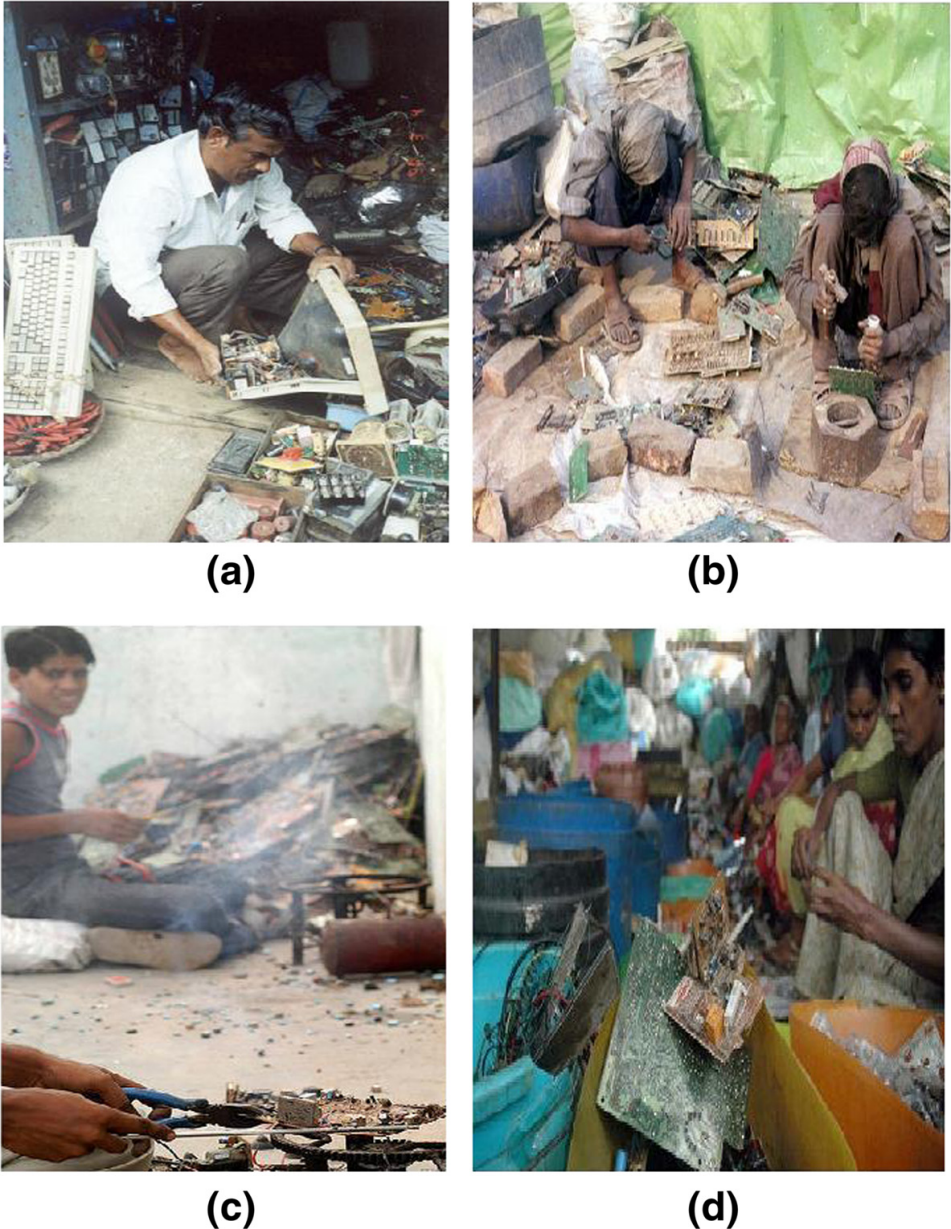
**E-waste recycling units in India. (a)**: E-waste dealer sorting through waste in Chennai. **(b)**: Children recycling toxic e-waste with their bare hands, Delhi. **(c)**: Children extract copper from discarded computer parts. New Delhi. **(d)**: Women working in recycling toxic e-waste with their bare hands.

The Figure 3(a), (b), (c) and (d) shows how poor people, women and children are used in the E-waste recycling units in India.

### Basel convention - framing of the law

The incident that led to the creation of the Basel Convention was the Khian Sea waste disposal, in which a ship carrying incinerator ash from the city of Philadelphia in the US, after having dumped half of its load on a beach in Haiti, was forced away, sailed for many months changing its name several times unable to unload its cargo in any port, and ended up dumping much of it illegally at sea. The Basel Convention on the Control of Transboundary Movements of Hazardous Wastes and their Disposal is an International treaty, designed to reduce the movements of hazardous waste between nations, and specifically to prevent dumping of hazardous waste from developed to less developed countries (LDC) [22-26]. The Convention was opened for signature on March 22, 1989, and entered into force on May 5, 1992. This law has banned the export of hazardous waste to poorer countries since 1992, but the practice continues as pointed out by Chris Carroll (Woodell). Commonly, the term “bridging the digital divide” is used when old WEEE are exported to developing countries and they are often labeled as “second-hand goods” since the export of reusable goods is allowed. On the other hand, most WEEE that does work on arrival only have a short second life and/or are damaged during transportation. The main objectives of the Basel Convention are:

• Minimize the generation of hazardous waste.

• Dispose of hazardous wastes within the country of generation effectively in an environmentally sound manner.

• Establish enhanced controls on exports and imports of hazardous waste.

• Prohibit shipments of hazardous wastes to countries lacking the legal and technical capacity.

### Recycling practice

Recycling faces a number of challenges, including dealing with hazardous materials such as cathode ray tube (CRT) glass and finding markets for flame-retardant plastics. Furthermore, at present no technology exists for recycling certain EEE in an environmentally friendly manner. In 2005, more than 2 million tons of E-waste were generated in the US alone, but only 17 to 18 percent of that was collected for recycling, informed by the Environmental Protection Agency(EPA) and the rest, more than 80 percent, was disposed of, largely in local landfills. The hazardous materials in E-waste can leaches out from the landfills into groundwater and streams, and if the plastic components are burned, dioxins are emitted into the air**.** Moreover, it is estimated that 50–80 percent of the E-waste collected for recycling in the US is actually exported to developing countries, even though it is illegal in most of those countries to accept this toxic waste stream. Much of this illegally traded waste is going to the informal recycling sectors in many Asian and West African countries, where it is dismantled or disposed of using very primitive and toxic technologies [10].

In India, most of the recycling happens in the informal sector where poor people tear apart the different components with their bare hands and without wearing any safety gear. In many such yards people are using cable waste as fuel to cook food. In fact, people are being exposed to toxins 24 hours a day as they live, cook and sleep in the same place where waste being recycled. Though E-waste is being recycled in all metros in India, Delhi is where the illegal and dangerous practices of recycling are adopted. India has become the dumping ground of all kinds of waste from the developed countries. A report from Manufacturers’ Association for Information Technology (MAIT) indicates that 50,000 tonnes are being imported every year [26-31].

### Health and environmental impact of E-waste

Electronic products are a complex mixture of several hundred tiny components, many of which contain deadly chemicals. These chemicals are a strain on human health and the environment. Most of the components in electronic devices contain lead, cadmium, mercury, polyvinyl chloride (PVC), brominated flame retardants (BFRs), chromium, beryllium etc., TVs, video and computer monitors use CRTs, which have significant amounts of lead and the long term exposure to these substances can damage the nervous system, kidney and bones and the reproductive and endocrine systems and some of them are carcinogenic. These e-wastes will have long lasting effects on the environment, when improperly disposed (incinerated/land filled instead of recycling) with domestic waste, without any controls, can contaminate the soil, water and air. EEEs are made of a multitude of components, some containing toxic substances that have an adverse impact on human health and the environment if not handled properly. Often, these hazards arise due to the improper recycling and disposal processes used. It can have serious repercussions for those in proximity to places where e-waste is recycled or burnt. In general the electronic goods/gadgets are classified under three major heads:

• White goods: Household appliances

• Brown goods: TVs, camcorders, cameras

• Grey goods: Computers, printers, fax machines, scanners etc.

**Figure 4:**
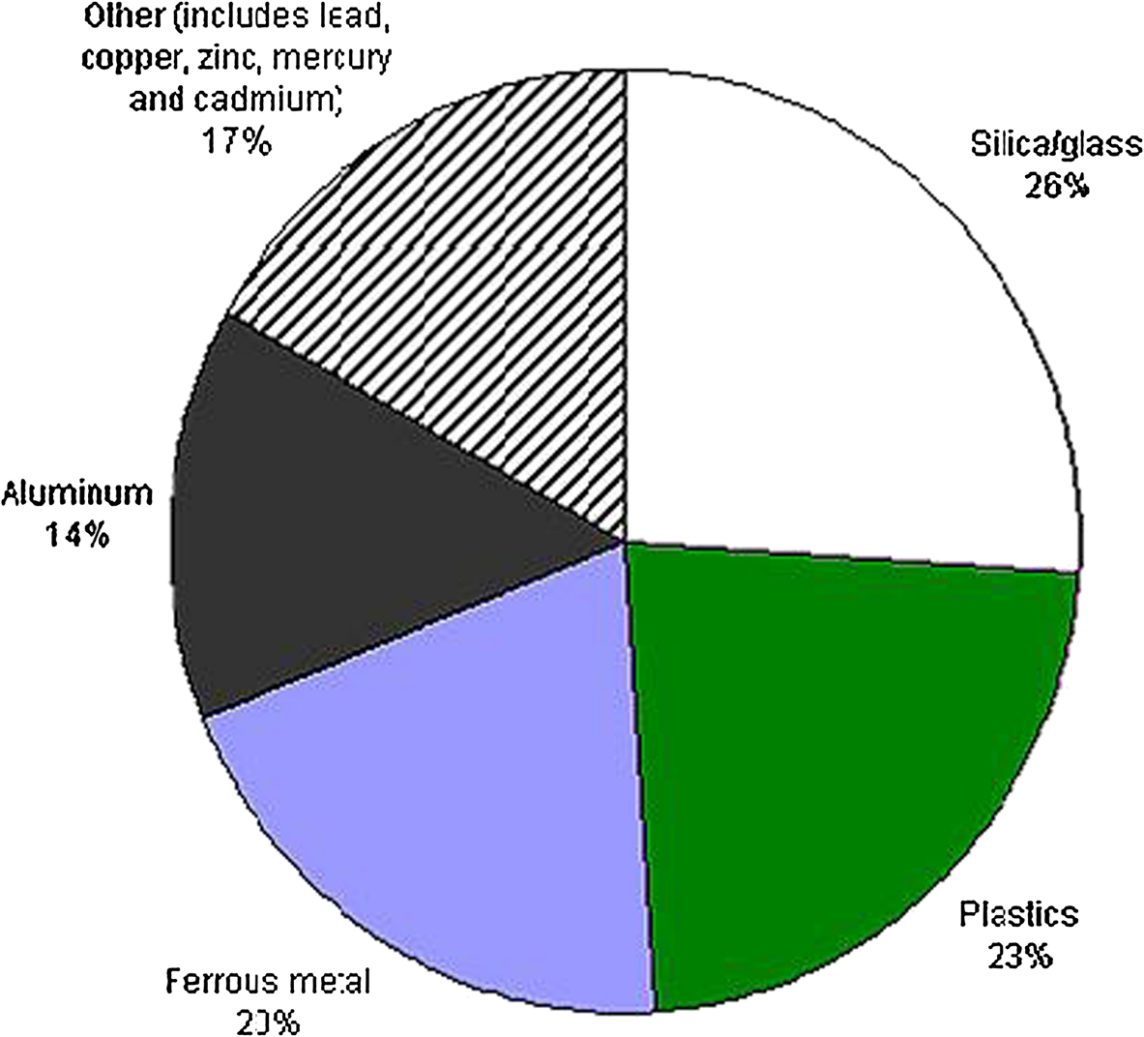
Personal computer material composition.

Waste from the white and brown goods is less toxic when compared to grey goods [29-36]. Even a personal computer contains highly toxic chemicals like lead, mercury, cadmium, etc., and its effect on health is shown and tabulated in Table 2 and Figure 4[32-37].

**Table 2:** Toxic metals present in various types of E-waste and their effects on humans

**Materials**	**Weight (%)**	**Recycling (%)**	**Location**	**Effects**
**Lead**	6.2988	5	Acid battery, CRT	Kidney failure, central and peripheral nervous systems, damage to the reproductive systems
**Cadmium**	0.0094	0	Battery, CRT, housing	Long term cumulative poison, Bone disease
**Mercury**	0.0022	0	Batteries, switches, housing	Chronic damage to brain, liver damage, causes damage to the central and peripheral nervous systems as well as the fetus
**Chromium VI**	0.0063	0	Decorative hardener, corrosion protection	DNA damage, lung cancer
**Plastic**	22.99	20	Computer mouldings, cablings	Generates dioxins and furans

### Chemical Leaching techniques of metals from E-waste

Chemical leaching involves leaching either by using acid or ligand supported complexation. Chemical leaching can also be performed by involving complexometry, where ligands get complexed with metals. Chemical leaching of metals from the E-waste can also be done by utilizing various inorganic-acids like; sulphuric acid (H_2_SO_4_), hydrochloric acid (HCl), and solution of H_2_SO_4_and HNO_3,_ Sodium hypochlorite (along with acid or alkali) can also be used for the recovery of precious metals like gold. Lee et al. (2009) used organic solvents for the extraction of heavy metals like Fe, Cu, Al, Ni, Au and Ag. Yang et al. (2011) studied chemical leaching of Cu present in waste PCBs with respect to its particle size, by utilizing treated shredded Cu particles of waste PCB with sulfuric acid and hydrogen-peroxide.

### Biological Leaching of E-waste

Sometimes, bleaching is a cost effective method in comparison to chemical leaching. Mainly acidophilic group of bacteria plays an important role in bioleaching of heavy metals from the wastes for instance *Acidithiobacillus ferrooxidans*, *Acidithiobacillus thiooxidans*, *Leptospirillum ferrooxidans*, and *Sulfolobus sp*. (Mishra and Rhee, 2010) Microorganisms are active in the formation and decomposition of various inorganic as well as organic matter on the earth’s crust. Bioleaching is based on the natural ability of microbes to transform solid metallic compounds to its solubility and extractable form. Autotrophic bacteria (e.g. *Thiobacilli sp*.), heterotrophic bacteria (e.g. *Pseudomonas sp*., *Bacillus sp*.) and heterotrophic fungi (e.g. *Aspergillus sp*., *Penicillium sp*.) are the three major groups of microbes involved in bioleaching of metals. Chemolithotrophs of iron- and sulfur-oxidizing nature (which grow autotrophically by fixing CO_2_ from the atmosphere) are the most important mineral-decomposing microbes [37-39] can be referred in Table 3.

**Table 3:** Metals present in various types of E-waste

**Types of electronic waste**	**Metal abundance (in %)**					**Metal present (in ppm)**			**Reference**
	**Pb**	**Ni**	**Al**	**Fe**	**Cu**	**Pd**	**Au**	**Ag**	**-**
**Printed wiring board**	2.6	2	7	12	16	-	0.04	-	[21,27,40]
**Printed circuit boards**	3	29	2	7	12	16		0.04	[25,40]
**Printed wiring board**	3	2	7	12	16		110	280	[33,35,40]
**PC board scrap**	1.5	1	5	7	20	110	250	1000	[26,40]
**PC main board scrap**	2.2	1.1	2.8	4.5	14.3	12.4	506	6.36	[29,40]
**TV board scrap**	1	0.3	10	28	10	10	17	280	[33,40]
**Mobile phone scrap**	0.3	0.1	1	5	13	210	350	1340	[21,40]
**Portable audio scrap**	0.14	0.03	1	23	21	4	10	150	[23]
**DVD player scrap**	0.3	0.05	2	62	5	4	15	115	[21,40]
**Calculator scrap**	0.1	0.5	5	4	3	5	50	260	[29]
**TV scrap**	0.2	0.04	1.2		3.8	27.1	27.1	27.1	[37,38,40]
**PC scrap**	6.3	0.85	14.17	20.47	6.93	3	16	189	[40]

Acidolysis, complexolysis, redoxolysis and bioaccumulation are the common mechanism involved in bioleaching [32]. At 40°C or less, mineral biooxidation processes involving the use of microorganisms are believed to be comprised of a consortium of gram-negative bacteria which includes iron- and sulfur- oxidizing *A. ferrooxidans*[35]. Some workers [35,40] reviewed rapidly growing microbial-based metal extraction industry, which are utilizing a diversity of microbes that can grow at variable temperatures, involving either rapid stirred-tank or slower irrigation technology to recover metals from their ores [41,42]. Microorganisms have a tendency to extract metals from its sulfide and or iron-containing ores and mineral concentrates. Iron and sulphide are microbially oxidized to produce ferric ion along with sulphuric acid, consequently these chemicals convert insoluble sulfides of metals such as copper, nickel and zinc to soluble metal sulfates that can be readily recovered from the solution. Pham and Ting (2009) extracted Au from E-waste by utilizing cynogenic-bacteria (Chromobacterium violaceum and *Pseudomonas fluorescens*) along with a pretreatment for bio-oxidation of E-waste by *A. ferrooxidans* (which specifically remove Cu leaving Au residues behind) [43]. Au and Ag can also be extracted employing various other microbes like *Acidithiobacillus sp, Leptospirillum sp*, *Ferromicrobium sp* and *Acidiphilium sp.* mineral-extraction is achieved both by ‘direct (contact) leaching’ using bacteria and ‘indirect leaching’ by ferric ion. However, the procedure for extraction is not clearly reported.

### Hybrid Technique for metal extraction

Generally biological leaching is a cost effective technique but time consuming, even the complete recovery of metal alone by biological leaching is not possible in most of the cases [44]. Chemical leaching on the other hand is comparatively rapid and efficient but it has its own environmental issues. If a hybrid technique is proposed, involving combination of chemical (safer chemicals) and biological leaching, so that both will complement each other for an effective and improvised method for metal extraction.

## Conclusion

India is placed among the other global nations which have generated more E-waste in quantity and especially urban India needs an urgent approach to tackle this issue. Technical and policy-level interventions, implementation and capacity building and increasing the public awareness can convert this challenge into an opportunity to show the world that India is ready to deal with future problems and can set global credible standards concerning environmental and occupational health. Microsoft has created a Vision of 2020 that neutralizes the appeal of physical devices, regulating them to the background and it is not waiting for consumers to determine the future of electronics. By doing so, Microsoft would create a future where very little e-waste will be generated because the devices serve simply to facilitate our engagement in the world around us and it’s a new idea as well. HP has developed a safe cleaning method for chips using carbon-di-oxide as a substitute for hazardous solvents. Even in 1998 IBM introduced the first computer that uses 100% recycled resins in all major plastic parts. Toshiba is working on a modular upgradeable and customizable computer to cut down on the amount of product obsolescence.

Recycling is the key to reduce the E-waste and it has environmental benefits at every stage in the life cycle of a computer product, from the raw material from which it is made to its final disposal. Aside from reducing greenhouse gas emissions, which contribute to global warming, recycling also reduces air and water pollution associated with making new products from raw materials. By utilizing used, unwanted, or obsolete materials as industrial feedstock or for new materials or products, we can do our part to make recycling work. Though Government of India is signatory to the Basal Convention, there is no clear policy and control of Transboundary Movement of Hazardous wastes and their disposal. Draft Hazardous Materials (Management, Handling and Transboundary movement) Rules, 2007 (dated, September 28, 2007), is a part of the Environment Protection Act, 1986 is already enacted to support the control of hazardous and toxic waste movements.

Future efforts to minimize illegal dumping will undoubtedly include a combination of aggressive legislation, new technological solutions, and increased public awareness through more education on e-Waste. Chemical and biological leaching has their own merits and demerits and there could be various technical, economic and environmental reasons for choosing one process over the other. Hybrid methodology has the potential to overcome the problems associated with chemical and biological extraction techniques for the metals present in E waste. This strategy can provide new and emerging area of metallurgy which may facilitate the extraction of metals present in trace quantity from their ores. As a result we should know the ways and means of disposing the waste with the help of the available or new technology for a convincing betterment of our environment.

## Consent

Consent was obtained while photographing the individuals.

## Abbreviations

Al: Aluminium; Au: Gold; Ag: Silver; BFR: Brominated flame retardants; CO2: Carbon di-oxide; CPU: Central processing unit; CRT: Cathode ray tube; Cu: Copper; EEE: Electrical and electronics equipment; EPA: Environmental protection agency; EU: European union; Fe: Iron; HCl: Hydrochloric acid; HNO3: Nitric acid; H2SO4: Sulphuric acid; IT: Information technology; LDC: Less developed countries; MAIT: Manufacturers’ association of information technology; PC: Personal computer; PCB: Printed circuit board; TV: Television; US: United States; WEEE: Waste electrical and electronics equipments.

## Competing interests

The authors declare that they have no competing interests.

## Authors’ contributions

SN participated in the design of the study and supervised the work. MS and RC did the analyses, and/or interpreted the analyzed results. SN wrote the initial draft and revised the paper critically for important intellectual content and compiled the work in accordance to journal format. All authors have read and approved the final manuscript.

## Author information

1. Professor and Head (Academics), Department of Civil Engineering, Saveetha School of Engineering, Saveetha University, Chennai, India.

2. Research Scholar, School of Biosciences and Technology, VIT University, Vellore, India.

3. Professor, Dean, School of Biosciences and Technology, VIT University, Vellore, India.
